# A case–control study of breast cancer risk and ambient exposure to pesticides

**DOI:** 10.1097/EE9.0000000000000070

**Published:** 2019-09-30

**Authors:** Carrie Tayour, Beate Ritz, Bryan Langholz, Paul K. Mills, Anna Wu, John P. Wilson, Kaveh Shahabi, Myles Cockburn

**Affiliations:** a Department of Preventive Medicine, Keck School of Medicine, University of Southern California, Los Angeles, California; b Los Angeles County Department of Public Health, Los Angeles, California; c Departments of Epidemiology and Environmental Sciences, Fielding School of Public Health, University of California, Los Angeles, California; d Department of Medicine, University of California, San Francisco, Fresno, California; e Spatial Sciences Institute, University of Southern California, Los Angeles, California.

**Keywords:** Breast cancer, Pesticides, Geographical Information Systems, Exposure Assessment

## Abstract

**Background:**

While the estrogenic properties of certain pesticides have been established, associations between pesticide exposure and risk of breast cancer have been inconsistently observed. We investigated the relation between pesticide exposure and breast cancer risk using methods capable of objectively assessing exposure to specific pesticides occurring decades before diagnosis.

**Methods:**

A case–control study was conducted to evaluate the risk of postmenopausal breast cancer associated with historic pesticide exposure in California’s Central Valley, the most agriculturally productive region in the United States where pesticide drift poses a major source of nonoccupational exposure. Residential and occupational histories were linked to commercial pesticide reports and land use data to determine exposure to specific chemicals. Cases (N = 155) were recruited from a population-based cancer registry, and controls (N = 150) were obtained from tax assessor and Medicare list mailings.

**Results:**

There was no association between breast cancer and exposure to a selected group of organochlorine pesticides thought to have synergistic endocrine-disrupting potential; however, breast cancer was three times as likely to occur among women exposed to chlorpyrifos compared with those not exposed, after adjusting for exposure to other pesticides including organochlorines (OR = 3.22; 95% CI = 1.38, 7.53).

**Conclusions:**

Organophosphate pesticides, such as chlorpyrifos, have rarely been evaluated in studies of breast cancer risk. Additional research is needed to confirm these findings and to better understand the underlying mechanisms given that chlorpyrifos has been detected in local air monitoring at levels of concern for residents living in the agricultural regions where it is used.

**What this study adds**

Exposure to pesticides might contribute to breast cancer development, but estimating adult cancer risks associated with previous cumulative exposures presents methodologic challenges, requiring accurate measurements over many decades. We have constructed a comprehensive pesticide exposure assessment using historic pesticide data and geocoded location histories in a case–control study of pesticides and breast cancer. This study suggests that pesticides other than organochlorines, such as the organophosphate chlorpyrifos, may be important for breast cancer risk and that additional research is needed to improve etiologically relevant measures of exposure to protect people who are unknowingly exposed to these chemicals.

## Introduction

Because lifetime estrogen exposure is a key factor in breast cancer development, exposure to endocrine-disrupting pesticides might contribute to breast cancer development.^1–3^ While the estrogenic properties of some pesticides have been established,^4–6^ results from previous studies of pesticide exposure on breast cancer risk are conflicting: some studies show a positive association,^7–11^ while others are null.^12–20^

Many previous epidemiologic studies relied upon self-reported exposure based on pesticide usage, occupation, or living on a farm.^11,14,17–20^ Aside from a cohort study which did find some associations for specific pesticides,^7,13^ the majority of these studies grouped together pesticides with varying toxicologic effects, likely leading to nondifferential exposure misclassification and reported null effects that may have obscured associations with specific chemicals. Other studies used ecologic designs,^16^ spatial regression,^8,9^ or proximity to aggregated pesticide data at only one residential location.^10,15,20^ Studies that use biomarkers to measure pesticide metabolites in serum samples taken near the time of cancer diagnosis may not reflect previous exposures that are most relevant for breast cancer etiology and do not reflect long-term exposure.^21^ There is a need for research methodologies that can reconstruct exposures occurring decades before diagnosis and evaluate pesticide-specific exposures on breast cancer risk.^22,23^

We conducted a case–control study of breast cancer risk from exposure to pesticides using a Geographical Information Systems (GIS)–based method that combines geocoded residential and occupational histories with state pesticide use reports and land use data^24^ in California’s highest-ranking counties (Fresno, Tulare, and Kern) for agricultural density and commercial pesticide use in the United States.^25^ In highly agricultural regions, pesticide drift from neighboring application sites presents a major source of nonoccupational exposure.^26–30^ We evaluated a group of structurally and toxicologically similar^31^ organochlorine pesticides with known estrogenic effects that are most likely related to breast carcinogenesis because of their ability to accumulate in adipose tissue and potential to act synergistically (aldrin, chlordane, dicofol, dieldrin, endosulfan, lindane, methoxychlor, and toxaphene).^3,5,32–36^ We also assessed breast cancer risk from exposure to three commonly applied pesticides in the region (chlorpyrifos, diazinon, and 1,3-dichloropropene) detected at levels of concern to human health in air monitoring conducted by the California Department of Pesticide Regulation (CDPR) in 2006 in a Fresno County farming community.^37^ Because no regulatory ambient air standards exist for most pesticides, CDPR developed health-based screening levels for 35 pesticides and found that diazinon exceeded its screening level, chlorpyrifos approached its screening level and was frequently detected, and 1,3-dichloropropene exceeded its cancer potency value.

## Materials and methods

### Participant recruitment

Cases were recruited from among women with histologically confirmed breast cancer diagnosed in 2007–2008 in the counties of Fresno, Tulare, or Kern from the Cancer Registry of Central California (CRCC), 55–74 years of age, and of non-Hispanic white ethnicity. From 2011 to 2013, cases were recruited by telephone. Among the 328 eligible cases we attempted to recruit, 10 were deceased and four too ill, 123 refused to participate, and we were unable to contact 32. Cases were excluded if they were premenopausal (N = 0) because postmenopausal breast cancer is more likely of hormone-related origin, reported Hispanic ethnicity (N = 2), or had been diagnosed with ovarian, uterine, or other female reproductive cancers before their diagnosis of breast cancer (N = 2). To match control selection criteria (below), cases were excluded if they had not lived in California for at least 5 years (N = 0) or had Parkinson’s disease (N = 0). A total of 155 participants with breast cancer completed the study.

Controls were obtained from another population-based case–control study we conducted in the same geographic area between 2001 and 2011 examining the risk of Parkinson’s disease. Controls lived in California for at least 5 years before the study were at least 35 years old, resided in Fresno, Tulare, and Kern counties, and did not have Parkinson’s disease. Details of control selection are provided elsewhere.^38–40^ Controls were recruited from Medicare listings, mailings to a random selection of tax assessor parcel addresses using Internet searches and marketing companies to identify contact information, and from 2009, additional participants were enrolled in person during home visits to randomly selected households. The overall response rate among controls was 46%.^40^

From the controls enrolled in the Parkinson’s disease study, we selected postmenopausal women 55–74 years of age of non-Hispanic white ethnicity (N = 208). After excluding women who had been diagnosed with breast cancer (N = 20) ovarian, uterine, or other female reproductive cancers (N = 9), and women who had opted to complete a shortened questionnaire without lifetime residential and occupational histories (N = 29), there were 150 participants included as controls in these analyses.

### Source of exposure data

All controls (N = 150) and the majority of cases (N = 111) were interviewed over the telephone, with an additional 44 cases opting to complete a mailed questionnaire with follow-up by telephone to clarify or complete responses. All study participants were mailed a timeline to complete their historic residential and occupational workplace information (addresses and dates) before their telephone interviews. All historic residential and occupational workplace addresses were geocoded using Texas A&M GeoServices geocoder (available at http://geoservices.tamu.edu) and manually resolved to rooftops or by using additional information from participants such as cross streets and landmarks to more precisely identify a location^41^ and improve accuracy of rural locations in particular.^42^ We noted the level of “certainty” of each geocoded location and considered addresses to have high geocode certainty if geocoded to the centroid of a building, parcel, nearest parcel, street, or street intersection. Addresses geocoded to the centroid of a zip code, city, county, or state, or those that were unable to be geocoded were considered to have low geocode certainty.

### Historic pesticide exposure assessment

Historic pesticide exposures were determined from our GIS-based method that combines state-reported pesticide use data, land use surveys, and geocoded addresses to provide estimates of pesticide exposure within a 500-m buffer around residential and occupational locations. These methods have been described in detail elsewhere.^24,40^ Briefly, historic pesticide exposures are estimated by linking residential locations with California Pesticide Use Reporting (PUR) data,^43^ containing the name of the pesticide active ingredient, the pounds applied, the crop and acreage of the field to which it was applied, the application method, and the date and location. These data were enhanced with land use data from countywide surveys conducted every 6–10 years by California’s Department of Water Resources (DWR), Division of Planning and Local Assistance,^44^ to refine the geographical resolution to the crop level as previously reported.^24,45^

Historic ambient exposure to specific pesticides of interest was calculated by summing the annual density (total pounds of a pesticide’s active ingredients applied per acre) of applied pesticide within a 500-m buffer around each residential and occupational location.^24^ This buffer distance was chosen based on studies that found measurable concentrations of pesticides from commercial pesticide application detectable in household dust of neighboring homes.^46–48^

### Source of covariate data

Telephone interviews were conducted to obtain the covariates age (in years), ever lived on a farm (yes or no), ever worked on a farm (yes or no), education (in years), age at menarche, age at menopause (natural or surgical), number of births (including stillbirth), oral contraceptive use (in years), menopausal hormone therapy use (in years) by type (estrogen only, progesterone only, estrogen plus progesterone, or a mixture of treatments), ever smoked (current, former, and never), ever consumed alcohol at least once a week (yes or no), and vigorous physical activity (defined as the number of hours of strenuous or moderate activity per week). Weight (pounds) and height (feet and inches) at the time of diagnosis or interview were used to calculate body mass index (kg/m^2^). Neighborhood socioeconomic status (SES) was based on the residential address at the time of diagnosis for cases or at the time of interview for controls using income and occupation information obtained from the 1990 US census data at the block group level and categorized into a quintile score.^49^

Participants were asked if they ever personally applied pesticides (yes or no) inside their homes, outdoors in their yards or gardens, on their pets, and whether they had ever hired a professional to spray or fumigate, as well as whether they had ever worked on a farm or with pesticides or fertilizers. To identify occupational pesticide exposure based on self-reported data, other studies have used job-exposure matrices,^50^ but only 26 cases and 43 controls reported farming so occupational exposure was based on self-reported “ever” or “never” worked on a farm or worked with pesticides.

### Statistical analyses

Unconditional logistic regression was used to estimate ambient pesticide exposure on the risk of breast cancer in postmenopausal women. Odds ratios (ORs) and 95% confidence intervals (CIs) were calculated for study participants exposed to specific pesticides compared with those not exposed. An individual was considered exposed to a particular pesticide when the pounds per acre of applied pesticide within the buffer area were greater than zero during the period from 1974 until the year of diagnosis for cases and the year of interview for controls. We chose 1974 as the start of our exposure assessment to include all years with complete pesticide information recorded by the State. To account for time between exposure and the development of the disease, we conducted sensitivity analyses excluding 10 and 20 years before diagnosis.

All analyses were adjusted for established breast cancer risk factors including age (continuous), SES (quintiles 1 lowest to 5 highest), body mass index (<25, 25–29, or ≥30 kg/m^2^), age at menarche (<12, 12, or >12 years), age at menopause (<45, 45–54, or ≥55 years), number of births (0, 1, 2, or ≥3), oral contraceptive use (none, 1–4 years, or ≥5 years), menopausal hormone therapy use (none, estrogen only, progesterone only, estrogen plus progesterone, or a mixture of treatments), and ever consume alcohol at least weekly (yes or no), as well as the number of years lived in Fresno, Tulare, and Kern counties during the exposure assessment time period (continuous). Other factors, including the year of diagnosis, education, ever smoked, vigorous physical activity, ever lived or worked on a farm, and ever personally applied pesticides inside their home, outdoors in their yard or garden, or on their pets, and ever hired a professional to spray or fumigate, were evaluated as potential confounders and were included in the final models if they changed the estimates by >10%. All analyses were conducted using SAS, version 9.3 software (SAS Institute, Inc., Cary, NC).

For the years residential histories had missing location data due to incomplete recall of addresses or addresses that could not be found, we imputed exposures using the average exposures during all years with data for each person.^51^ Gaps in workplace histories where women did not report addresses because they were unemployed, at home caring for children, retired, or disabled were imputed with the participant’s residential exposure for that respective time frame (assuming that they most likely resided at home during typical work hours). Pesticide exposure could not be identified for addresses outside of California because the exposure model includes only California PUR data, but participants reported whether any non-California addresses were on farms. We assessed the influence of missing data due to recall and locations outside of California by examining the change in our risk estimates after excluding missing data and conducted sensitivity analyses that included only women who lived in California for at least 30 years between 1974 and their year of diagnosis or interview. We also looked the potential impact of migration in our study by evaluating the influence of demographic factors (age and SES) and disease status among women who moved during our exposure assessment to women who resided in California for at least 30 years and to women who resided in Fresno, Tulare, and Kern counties for at least 30 years.

The institutional review boards at the California Health and Human Services Agency and the University of Southern California approved the study protocol for cases participating in this study. The institutional review board at the University of California, Los Angeles, approved the study protocol for controls used in this study. Informed consent was obtained for all participants.

## Results

Cases and controls appeared similar in terms of established breast cancer risk factors such as age, socioeconomic status, education, body mass index, age at menarche, age at menopause, number of births, menopausal hormone therapy use, and vigorous physical activity (Table 1). Cases were more likely to have used oral contraceptives for 5 years or longer (OR = 1.17; 95% CI = 0.70, 1.97). Cases were half as likely to be current smokers (OR = 0.45; 95% CI = 0.20, 1.03) but were more likely to consume alcohol at least weekly compared with controls (OR = 1.74; 95% CI = 1.09, 2.79). Cases were more likely to have lived in Fresno, Tulare, and Kern Counties at least 30 years since the start of our exposure assessment in 1974 and to have lived in California at least 30 years (OR = 1.59; 95% CI = 1.01, 2.51; and OR = 2.52; 95% CI = 1.28, 4.97, respectively). Cases were half as likely to have lived and worked on a farm (OR = 0.44; 95% CI = 0.23, 0.83) (Table 2) or to have worked with pesticides compared with controls, but only three cases and seven controls reported working with pesticides. Cases were more likely to have applied pesticides in their yards or gardens than controls (OR = 1.75; 95% CI = 1.08, 2.82).

**Table 1:**
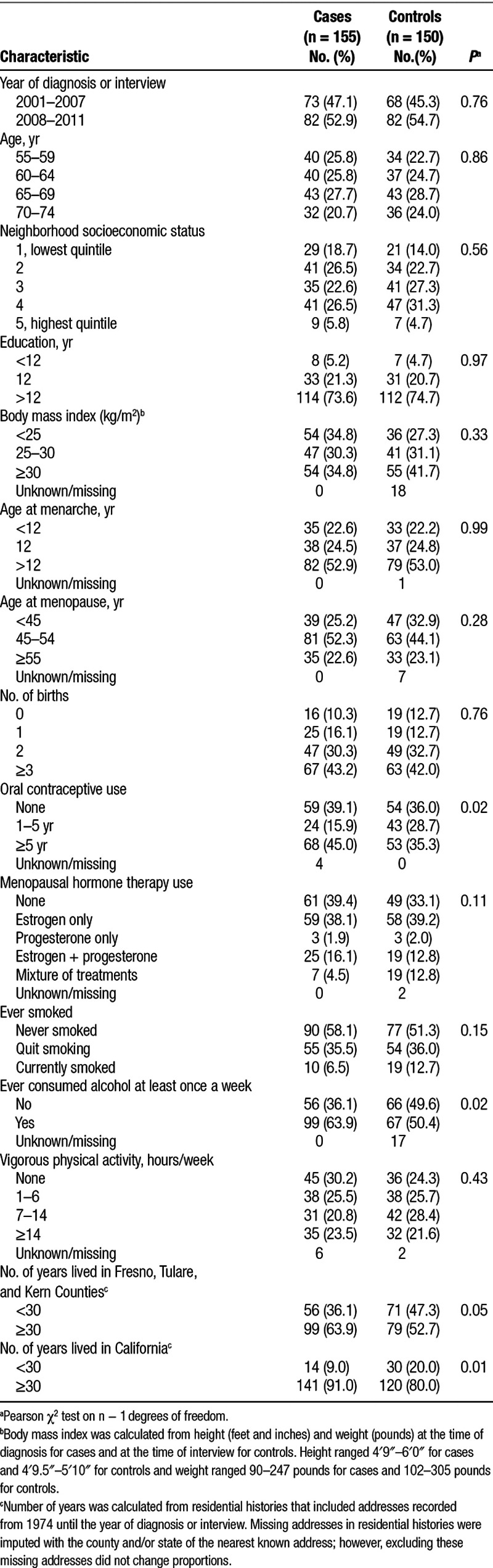
Comparison of selected characteristics in breast cancer cases (diagnosed in 2007–2008) and population-based controls (2001–2011), in Fresno, Tulare, and Kern Counties

**Table 2:**
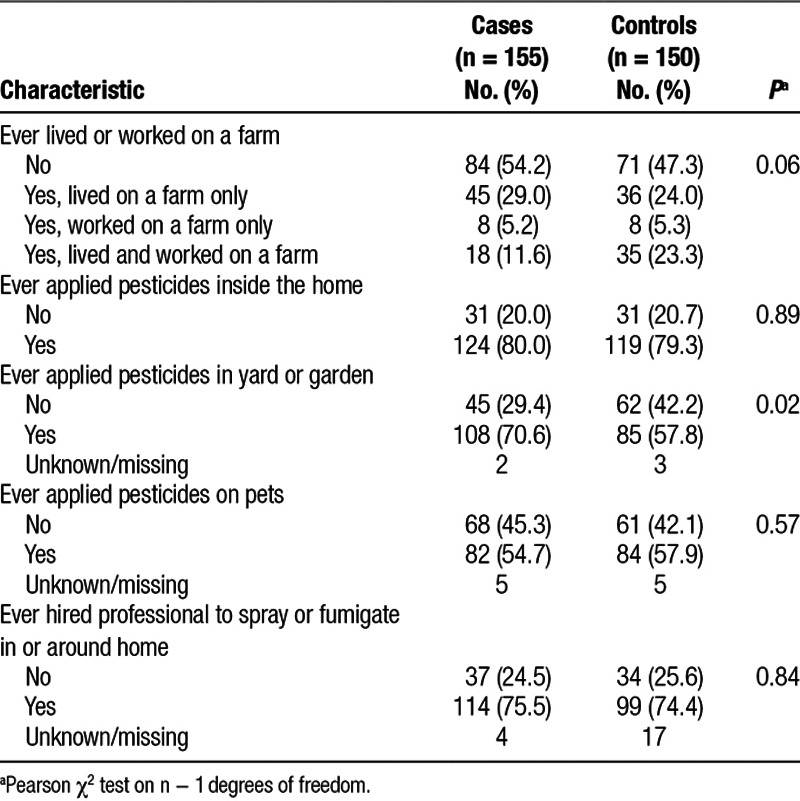
Comparison of self-reported living or working on a farm and personal pesticide use among breast cancer cases (diagnosed in 2007–2008) and population-based controls (2001–2011), in Fresno, Tulare, and Kern Counties

When assessing ambient pesticide exposure, the prevalence of exposure at residences and workplaces for the selected group of organochlorines, chlorpyrifos, and diazinon were over 40% among cases and controls (Table 3). After adjusting for breast cancer risk factors, the number of years lived in Fresno, Tulare, and Kern counties, vigorous physical activity (because its inclusion changed the estimates by 19.1%), and exposure to the other pesticides, breast cancer was three times as likely to occur among women exposed to chlorpyrifos at both residences and workplaces compared with those not exposed at either location (OR = 3.22; 95% CI = 1.38, 7.53). Associations more moderate in magnitude were observed between breast cancer and exposure to the group of organochlorine pesticides and to diazinon; however, after adjusting for exposures to other pesticides, in particular chlorpyrifos, the associations were null (OR = 0.98; 95% CI = 0.42, 2.28; and OR = 0.81; 95% CI = 0.35, 1.84, respectively). The vast majority of organochlorine pesticide applications during our exposure assessment were dicofol and endosulfan; and restricting the analyses to only these two organochlorines did not qualitatively change our estimates. There was no increased breast cancer risk for exposure to 1,3-dichloropropene. Results that excluded exposures occurring 10 or 20 years before diagnosis or interview did not qualitatively change the risk estimates. Risk estimates were slightly attenuated for exposure to chlorpyrifos (OR_10 year_ = 2.78; 95% CI = 1.20, 6.43; and OR_20 year_ = 3.13; 95% CI = 1.30, 7.52) and remained close to the null for exposure to organochlorines, diazinon, and 1,3-dichloropropene, after accounting for 10- and 20-year latency periods.

**Table 3:**
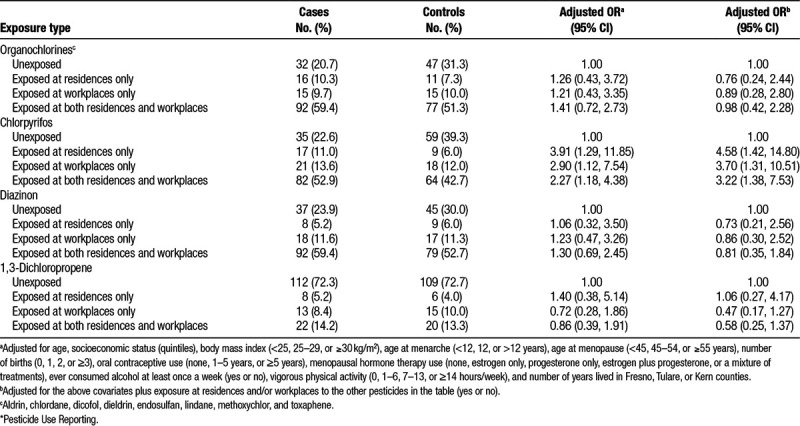
Measures of association between breast cancer and ambient exposure to selected pesticides based on residential and occupational address histories among women in Fresno, Tulare, and Kern counties using linked PUR* data for 1974–2011

Among those reporting that they never lived on a farm, 43.9% of cases and 40.0% of controls were exposed to pesticide drift from one of the selected pesticides at their residence. Among those who reported never working on a farm, 71.6% of cases and 54.7% of controls were exposed to pesticide drift from one of the selected pesticides at their workplace.

Excluding the subjects with substantial missing information in their residential histories did not qualitatively change our estimates. There were three cases and four controls with more than one third of their residential timelines missing. The majority of participants had all residential locations within California during the timeframe of interest (82.0% of cases and 72.7% of controls). Few participants reported that their non-California residences were on farms (three cases and five controls). Restricting analyses to include only those who had lived in California for at least 30 years during our exposure assessment time frame increased estimates for exposure to chlorpyrifos at both residences and workplaces (OR = 3.98; 95% CI = 1.48, 10.72), while estimates remained null for exposures to the other pesticides (data not shown). Participants who migrated out of California or migrated from Fresno, Tulare, and Kern counties during the time period of our exposure assessment did not differ from participants who did not migrate by demographic factors (age and SES) or by disease status (data not shown).

## Discussion

This population-based study examined historic and chemical-specific effects of hormone-related pesticides that are plausibly related to breast cancer in a region of intense agricultural production. We did not observe an association between breast cancer risk and exposure to a group of organochlorines after adjusting for coexposures to other commonly applied pesticides. Conversely, we observed that breast cancer was three times as likely to occur with exposure to the organophosphate chlorpyrifos, one of the three pesticides detected in air monitoring studies at levels of concern to public health (OR = 3.22; 95% CI = 1.38, 7.53). The majority of epidemiologic studies involving pesticides and breast cancer risk have focused on organochlorines, but the increased risk of breast cancer with exposure to the organophosphate chlorpyrifos became stronger after adjusting for exposure to other pesticides including organochlorines.

Previous studies examining exposure to organochlorines have not considered other kinds of pesticides such as chlorpyrifos that may be highly correlated with organochlorines and are driving the breast cancer association. When assessing ambient pesticide exposure, it is usually difficult to distinguish the effects of specific chemicals because applications may be correlated and people can be exposed to multiple chemicals (as is the case with other kinds of toxic air pollutants). For example, among the controls in our study, 54.0% were exposed to both the organochlorines and chlorpyrifos, 24.7% were unexposed to either, and only 21.3% were exposed to one but not the other. Although our chemical-specific exposure model evaluated risk from a group of organochlorine pesticides with potential for synergistic effects, more research is needed to understand possible correlations among applications of pesticides.

Findings from this study suggest that exposure to pesticides other than organochlorines may also affect breast cancer risk. These results support the findings from a large cohort study of farmers’ wives in the Agricultural Health Study, which reported that exposure to chlorpyrifos was one of the pesticides driving a possible breast cancer association (OR = 1.40; 95% CI = 1.0, 2.0) but did not find any associations with entire classifications of organochlorine or organophosphate pesticides.^7^ The prevalence of pesticide exposure from ambient sources in this study was over four times the prevalence of self-reported pesticide usage by farmer’s wives, and self-reported exposures do not account for exposures that people may not be aware of but are routinely exposed to, which is often the case with pesticide drift. According to our GIS-based exposure model, among participants in our study who never lived on a farm, 40% or more lived in residences that were within 500 feet of commercial pesticide applications and over 50% were potentially exposed by proximity to their workplace locations.

Evidence is mounting for pesticides besides established organochlorines to act as endocrine disruptors that can increase the risk of breast cancer, particularly for chlorpyrifos. Chlorpyrifos is weakly estrogenic^4,5,52^ and antiandrogenic.^53,54^ It can affect hormone pathways as an aryl hydrocarbon receptor agonist^55^ and induce proliferation of estrogen-dependent breast cancer cells in vitro.^56^ At low doses, chlorpyrifos promotes mammary tumor development and alters mammary gland hormone balance in vivo.^57,58^ More toxicologic research is needed to understand its mechanistic potential with regard to breast cancer risk. The findings from this study, however, support the 2012 regulations that restrict aerial pesticide application of chlorpyrifos to reduce the potential for exposure through pesticide drift.^59^

Both chlorpyrifos and diazinon were voluntarily phased out for residential uses in 2000 and 2001, but still used in agriculture.^60,61^ Although chlorpyrifos and diazinon are both organophosphate pesticides, and as many women were exposed to diazinon as to chlorpyrifos in our study, we did not find an association between diazinon and breast cancer risk after adjusting for exposure to chlorpyrifos. Reasons for this could be due to differing toxicities because chlorpyrifos is cytotoxic at far lower concentrations than diazinon in vitro^62^ or due to different exposure potential indicated by the finding that commercial application of chlorpyrifos but not diazinon was significantly correlated with measurements in household dust, even though diazinon was used twice as often.^28^

We did not observe an association between breast cancer and 1,3-dichloropropene; however, the prevalence of exposure was low (14.2% of cases and 13.3% of controls exposed at both residences and workplaces). The pesticide 1,3-dichloropropene is a respiratory carcinogen, but its role as a breast carcinogen is unknown.^63^

Strengths of this study include GIS-based exposure design that constructs exposure occurring over decades based on individual residential and occupational histories, while controlling for established breast cancer risk factors. Previous studies have estimated pesticide exposure by proximity to applications or crops using addresses at the time of cancer diagnosis with limited information on potential confounders.^8,9,16,19^ Our risk estimates based on a single residential address at diagnosis or interview were null for all pesticides considered in these analyses, including exposure to chlorpyrifos (OR = 1.21; 95% CI = 0.56, 2.61), and may underestimate actual risk because they do not account for exposures occurring at workplaces. Only one previous study conducted in Cape Cod, MA, collected residential histories to assess GIS-based proximity to pesticide applications and found no associations; however, the prevalence of pesticide exposures was much lower than observed in our study, and exposures were grouped by land use type instead of by specific pesticide of interest.^12^

Exposure in this study was based on reported address histories rather than self-reported pesticide usage, reducing the potential for recall bias. A study conducted in Australia found that the association between breast cancer risk and self-reported “noticing of pesticide spray drift” was strongly confounded by participants’ belief in whether or not pesticides caused breast cancer (OR = 1.47; 95% CI = 1.15, 1.87 among believers and OR = 0.94; 95% CI = 0.51, 1.74 among nonbelievers).^64^

Our GIS-based method also reduces the potential for selection bias from differential participation as a result of concerns about exposures in the environment. Selection bias may still be a concern because cases were recruited from a population-based cancer registry while controls were obtained for another population-based study in the same location. Although we started interviewing controls earlier than cases, the median and distribution of dates of interviews are similar between cases and controls (Table 1). A time difference for enrollment of cases and controls could have a potential impact on the calculations of average annual exposure if there was a rapid change in the use or application of a pesticide that occurred near the years of diagnosis or interview, but the risk estimates were only slightly attenuated after accounting for 10- and 20-year latency periods. Controls were more likely to have lived or worked on a farm than cases and as a result would be expected to have higher likelihood of exposure to pesticides near their residences or workplaces, thus biasing our estimates toward the null; and yet, we still observed a strong association to one of the pesticides of interest. Cases, on the other hand, were more likely to have lived in Fresno, Tulare, or Kern counties longer than controls and, therefore, may have had more opportunity to be exposed to ambient pesticides in the region. We adjusted the analyses for the number of years lived in Fresno, Tulare, or Kern counties but still observed chemical-specific effects. Women who migrated during the exposure assessment did not differ from women who did not migrate by age, SES, or disease status; thus, migration is not likely to have differentially impacted our overall findings.

It is important to note that breast cancer cases participating in this study are of surviving cases. Participant cases were similar in age and SES to the population-based registry from which they were recruited but were less likely to have late-stage disease (data not shown). If there is a dose–response effect of pesticide exposure on severity of disease, then we are more likely to have recruited women with lower exposure, which would have resulted in an underestimation of the effects presented here. The geocode certainty of the historic addresses was similar for cases and controls for the period of our exposure assessment from 1974 until the year of diagnosis or interview. There were 92.3% of cases and 96.7% of controls residing 50% or more of the years during the time period at residential addresses having high geocode certainty and 78.1% of cases and 83.3% of controls working 50% or more of the years during the time period at workplace addresses having high geocode certainty. This suggests that the certainty of the geocoding is not likely to account for difference in the estimated effects.

## Conclusions

Estimating adult cancer risks associated with previous cumulative exposures present methodologic challenges for epidemiologic studies because it requires accurate measurements over lifetime and decades. The GIS-based methods presented here likely reduce exposure misclassification compared with estimates based on self-reported pesticide use. We have constructed a more comprehensive exposure assessment using historic data than has been done previously; however, a larger study is needed to confirm the chemical-specific associations we report and to examine different levels of exposure as well as potential dose–response relations.

This study suggests that pesticides other than organochlorines, such as chlorpyrifos, may be important for breast cancer risk and that additional research is needed to improve etiologically relevant measures of exposure to protect people who are unknowingly exposed to these chemicals in the air.
